# Effect of Pioglitazone on Endoplasmic Reticulum Stress and Autophagy Response in the Perivascular Adipose Tissue of Type 2 Diabetic Rats

**DOI:** 10.1155/ppar/9645836

**Published:** 2025-03-21

**Authors:** Erkan Civelek, Ecem Fatma Karaman, Sibel Özden, B. Sönmez Uydeş Doğan, Deniz Kaleli Durman

**Affiliations:** ^1^Department of Pharmacology, Faculty of Pharmacy, Istanbul University, Istanbul, Türkiye; ^2^Graduate School of Health Sciences, Istanbul University, Istanbul, Türkiye; ^3^Department of Pharmaceutical Toxicology, Faculty of Pharmacy, Biruni University, Istanbul, Türkiye; ^4^Department of Pharmaceutical Toxicology, Faculty of Pharmacy, Istanbul University, Istanbul, Türkiye

**Keywords:** autophagy, ER stress, HFD/STZ-induced T2DM, pioglitazone, PVAT

## Abstract

Perivascular adipose tissue (PVAT) plays a crucial role in vascular homeostasis. Recent studies in adipose tissue demonstrated that endoplasmic reticulum (ER) stress and autophagy are activated in Type 2 diabetes mellitus (T2DM), while the precise role of ER stress and autophagy in PVAT is unclear. We aimed to investigate the possible influence of pioglitazone on ER stress and autophagy response in PVAT of T2DM rats. T2DM was induced by high-fat diet/low-dose streptozotocin (HFD/STZ) in male Wistar rats (8–10 weeks), and pioglitazone (20 mg/kg/p.o.) was administered for 6 weeks. Changes in biochemical parameters (nonfasting glucose, total cholesterol, and triglyceride) were verified in blood samples. ER stress–related (*ATF4*, *CHOP*, and *GRP78*) and autophagy-related (*MAP1LC3B*/LC3-II, *BECN-1/*Beclin, and *SQSTM1*/p62) gene expression levels in thoracic PVAT were measured by RT-PCR. Pioglitazone treatment reversed the increased nonfasting glucose and triglyceride levels in T2DM. ER stress and autophagy responses were significantly increased in PVAT of T2DM rats. Pioglitazone increased ER stress–related *GRP78* gene expression while decreasing autophagy-related *MAP1LC3B* and *BECN-1* gene expression levels in T2DM. Interestingly, *SQSTM1* gene expression levels were increased by pioglitazone in the control and T2DM groups. The current study provides original findings regarding the effects of pioglitazone on ER stress and autophagy response in PVAT of HFD/STZ-induced T2DM rats. Pioglitazone treatment in T2DM increased *GRP78* and *SQSTM1* gene expressions, which both play a crucial role in adipocyte differentiation and adipogenesis, besides ER stress and autophagy. Further studies clarifying the adipogenic effect of pioglitazone on PVAT are needed for a better understanding of its effect on the vascular system.

## 1. Introduction

Perivascular adipose tissue (PVAT), the local adipose tissue layer surrounding blood vessels, releases a variety of adipokines and cytokines with different biological effects and is recognized as a key player in the vascular system [1]. The role of PVAT in the regulation of vascular tone has attracted attention since the very first report from Soltis and Cassis showing the anticontractile effect of PVAT [2]. The physiological function of PVAT has been shown to deteriorate in metabolic diseases such as obesity and Type 2 diabetes mellitus (T2DM) in terms of alteration in adipocytokine secretion profile and loss of anticontractile effects, which are collectively termed as PVAT dysfunction [3]. Increased inflammation and oxidative stress are determined to play a crucial role in PVAT dysfunction. We recently demonstrated that TNF-*α* protein levels are increased in PVAT of HFD/STZ-induced T2DM rats, but interestingly, this increased inflammatory profile was not accompanied with significant changes in the adiponectin and leptin levels as well as in the anticontractile effect of PVAT [4]. Moreover, previous studies have demonstrated that endoplasmic reticulum (ER) stress and autophagy are also activated in adipose tissue, particularly in T2DM, whereas the precise role of ER stress and autophagy in PVAT dysfunction remains largely unknown.

Several studies have reported that ER stress and autophagy responses are altered in adipose tissue in T2DM and related pathologies. In relation, autophagy was shown to be activated in subcutaneous adipose tissue [5] and omental adipose tissue [6] of the patients with T2DM. Likewise, ER stress was increased in the omental adipose tissues of patients with T2DM [6] and in the aortic PVAT of diabetic (db/db) mice [7]. To our knowledge, no study is available examining the role of autophagy in PVAT in T2DM.

Pioglitazone (Pio) is an oral antidiabetic drug that acts through peroxisome proliferator-activated receptor-*γ* (PPAR-*γ*) and has been shown to have beneficial effects on the cardiovascular system in preclinical and clinical studies [8–11]. Although the favorable effects of Pio on oxidative stress and inflammation are well defined [12, 13], the evidence on ER stress and autophagy is controversial. Several studies in in vivo and in vitro conditions displayed either an increase [14–18] or a decrease [13, 19–21] in ER stress. However, a study carried out on a human adipose cell line reported no change in ER stress by Pio [22]. On the other hand, an enhanced autophagic flux was documented in the liver of mice fed a high-fat diet (HFD) by Pio [23] while the accumulation of autophagy proteins was found to be reversed by Pio in the rostral ventrolateral medulla (RVLM) neurons of Wistar Kyoto rats fed a high-fructose diet [24]. However, it is unclear how Pio influences ER stress and autophagy in PVAT. Interestingly, our recent findings demonstrated a differentiation in the structural and inflammatory profile of PVAT in response to Pio treatment in HFD/STZ-induced T2DM, characterized by increased PVAT mass, adipocyte hypertrophy, and elevated TNF-*α* levels [4]. Therefore, exploring the effects of Pio on ER stress and autophagy responses will provide valuable insights into its role in PVAT remodeling and the associated vascular implications.

In this study, we aimed to investigate the possible influence of Pio on ER stress and autophagy response in PVAT of HFD/STZ-induced T2DM rats. For this purpose, ER stress–related (*ATF4*, *CHOP*, and *GRP78*) and autophagy-related (*MAP1LC3B*/LC3-II, *BECN-1*/Beclin, and *SQSTM1/*p62) gene expression levels are determined by real-time polymerase chain reaction (RT-PCR) in aortic PVAT following 6-week Pio (20 mg/kg/p.o.) treatment.

## 2. Material and Methods

### 2.1. Drugs and Chemicals

Rat standard chow and HFD (60% fat by calories) were purchased from MBD YEM TİC. AŞ., Türkiye. The composition of the HFD was as follows: 23.5% protein, 27.3% carbohydrate, and 34.3% fat, with adequate minerals and vitamins. Pio HCl (99.7% pure) was donated by Neutec Pharmaceuticals (Türkiye). Methylcellulose (MC) was purchased from Fluka (Switzerland) and streptozotocin (STZ) from Cayman Chemical (Michigan, USA). The Pio suspension was prepared by suspending Pio in 0.5% MC solution. STZ solutions were prepared daily by dissolving STZ in citrate buffer (pH = 4.5).

### 2.2. In Vivo Animal Studies

Eight- to 10-week-old Wistar Albino male rats (~250 g) obtained from Bezmialem Vakıf University Experimental Animals Laboratory were housed at 21°C ± 2°C, 45%–65% relative humidity, and 12 h/12 h light–dark cycle with free access to water and food. All procedures were carried out in the Experimental Animal Care and Research Unit of Istanbul University Faculty of Pharmacy (EDEHAB), according to the approval of the Local Ethics Committee of Animal Experiments of Istanbul University (IU-HADYEK) (06/04/2017, No. 133720). Randomisation was used to minimise potential confounders such as the animal/cage location, order of treatments, and measurements. In addition, blinding was used at all stages of the study by different investigators.

After 1 week of acclimatization, rats were weighed, and 20 rats were divided into four groups randomly: Group I: Control + MC, Group II: Control + Pio, Group III: T2DM + MC, and Group IV: T2DM + Pio, with 5 rats per each group. Random numbers were generated using the standard = RAND () function in Microsoft Excel. Rats in the control groups (Groups I and II) were fed standard rat chow, while rats in the T2DM groups (Groups III and IV) were fed a HFD (60% fat by calories) throughout the study. After feeding with HFD for 4 weeks, STZ (35 mg/kg) was administered intraperitoneally (i.p., 1 mL/kg) in the T2DM groups to induce diabetes (HFD/STZ-induced T2DM), while citrate buffer (solvent of STZ) was administered to the control groups, as described previously [4]. To confirm diabetes, nonfasting blood glucose levels were measured from the tail tips of rats by a glucometer 1 week after STZ administration. Rats with nonfasting blood glucose levels above 200 mg/dL were considered diabetic. Four weeks after the confirmation of diabetes, Pio (20 mg/kg) was administered to rats in Groups II and IV for a 6-week period via oral gavage (2 mL/kg), while MC, the solvent of Pio, was administered in Groups I and III. Following Pio administration, which corresponds to the end of the 15th week of in vivo experimentation, the thorax of rats was opened under thiopental (50 mg/kg/i.p.) anesthesia, blood samples were taken from the hearts, and rats were euthanized by exsanguination via cardiac puncture. Blood samples were immediately transferred to the laboratory and centrifuged at 3000 g for 10 min, and biochemical parameters (total cholesterol and triglyceride) were measured in serum samples. Thoracic aortas were isolated, and PVATs surrounding the aortas were detached. Thereafter, PVAT samples were snap frozen in liquid nitrogen and stored at −80°C until measurement of ER stress–related and autophagy-related gene expression levels.

### 2.3. Measurement of Biochemical Parameters

At the end of the 15th week of in vivo experimentation, nonfasting blood glucose levels were measured from the tail tips of rats via a glucometer (Accu-Chek Performa Nano, Roche, Basel, Switzerland). Total cholesterol and triglyceride levels were measured from serum samples via VetTest automated analyzer (Idexx, Maine, USA) by utilizing the enzymatic method. Tail tips were cleaned well with alcohol swabs after each sampling to avoid any risk of infection and inflammation.

### 2.4. Measurement of ER Stress–Related and Autophagy-Related Gene Expression Levels

Expression levels of ER stress–related *ATF4*, *CHOP*, and *GRP78* and autophagy-related *MAP1LC3B*/LC3-II, *BECN-1*/Beclin, and *SQSTM1/*p62 genes were measured in PVAT samples stored at −80°C. For this purpose, mRNAs were obtained from PVAT samples by using High Pure RNA Tissue Kit (Roche, Basel, Switzerland); then, cDNA was synthesized by using High-Capacity cDNA Reverse Transcription Kit (Thermo Fisher, Massachusetts, USA). Expression levels of related genes were measured by using the RT-PCR method via LightCycler 480 Instrument II (Roche, Basel, Switzerland). Specific primers for the genes of interest were designed by using Primer-BLAST [25]. The base sequence of the primers used was as follows: *ATF4* (forward CCC CAG GGT TTC TGT CTT CC, reverse TGG CGA GAG AAT CTG CCT TC), *CHOP* (forward CTG TTG GCA TCA CCT CCT GT, reverse AGT GTA CAA GCC CCT CTC CT), *GRP78* (forward CCA ATG ACC AAA ACC GCC TG, reverse TGG CTT TCC AGC CAT TCG AT), *MAP1LC3B/LC3-II* (forward GTT AAG CCC CTA CCA AGG CA, reverse AGG GAC TGT TTC CAG GGA CT), *BECN-1/Beclin* (forward CTC TCG TCA AGG CGT CAC TTC, reverse CAT TCT TTA GGC CCC GAC G), *SQSTM1/p62* (forward CTA GGC ATC GAG GTT GAC ATT, reverse CTT GGC TGA GTA CCA CTC TTA TC), and *β-actin* (forward CGT GCG TGA CAT TAA AGA G, reverse TTG CCG ATA GTG ATG ACC T).

Each of the samples used in RT-PCR experiments to determine mRNA expression values was studied in duplicate, and the average of the obtained cycle threshold (Ct) values was calculated and normalized using the internal control, *β-actin* (housekeeping gene), relative to the control group. The increase and decrease of the expression levels of genes were calculated by using the 2^−*ΔΔ*Ct^ method.

### 2.5. Statistical Analysis

All data are given as mean ± standard error of the mean (SEM), and “*n*” represents the number of rats. To compare rat weights, nonfasting blood glucose, serum total cholesterol, triglyceride, and gene expression levels between groups, one-way analysis of variance (ANOVA) followed by Tukey posttest was used. To check the normality of the distribution of datasets, Shapiro–Wilk's normality test was used and determined that all the mRNA expression data shows Gaussian distribution. *p* values < 0.05 were considered statistically significant. GraphPad Prism Software Version 5.00 was used for statistical analyses.

## 3. Results

### 3.1. Changes in Rat Weights

At the 15th week of in vivo experimentation, weight loss was observed in the T2DM + MC group compared to the control group (Control + MC), while Pio administration causes a significant weight gain in the T2DM group (T2DM + Pio vs. T2DM + MC, *p* < 0.05, *n* = 5), which was not observed in the control rats (Control + Pio vs. Control + MC, *p* > 0.05, *n* = 5) (Table 1).

### 3.2. Changes in Biochemical Parameters

Rats in the diabetes group had increased nonfasting blood glucose levels compared to the control group (T2DM + MC vs. Control + MC, *p* < 0.001, *n* = 5), while Pio administration in the T2DM group significantly decreased the nonfasting blood glucose levels when compared to the corresponding control group (T2DM + Pio vs. T2DM + MC, *p* < 0.001, *n* = 5) (Table 1).

Total cholesterol and triglyceride levels were increased in the diabetes group (T2DM + MC vs. Control + MC, *p* < 0.01 and *p* < 0.001, respectively, *n* = 5). Pio administration in the T2DM group did not influence total cholesterol levels (*p* > 0.05) while markedly decreased triglyceride levels compared to the corresponding control (T2DM + Pio vs. T2DM + MC, *p* < 0.01, *n* = 5) (Table 1).

### 3.3. ER Stress–Related Gene Expression Levels

**Figure 1:**
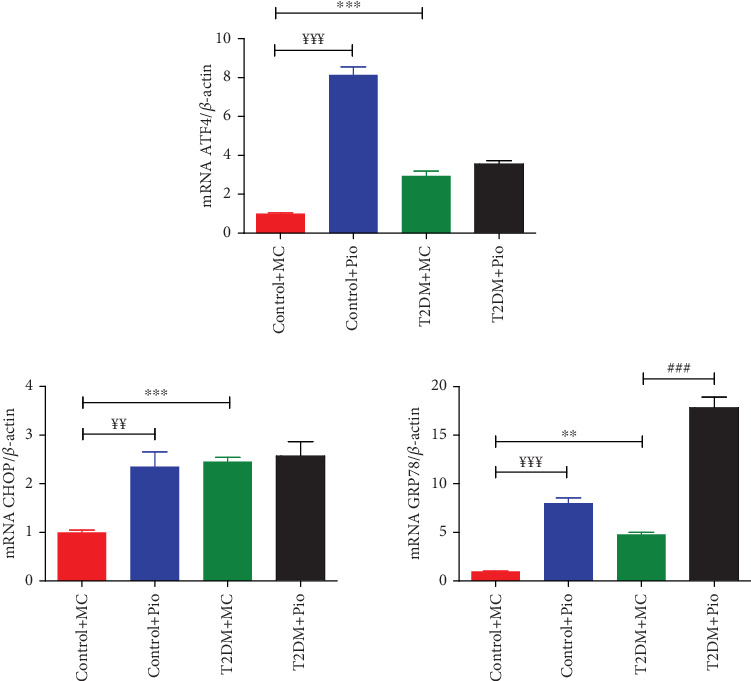
Comparison of ER stress–related *ATF4*, *CHOP*, and *GRP78* gene expression levels in PVAT of control (Control + MC and Control + Pio) and diabetic (T2DM + MC and T2DM + Pio) rats. ⁣^∗∗^*p* < 0.01, ⁣^∗∗∗^*p* < 0.001 T2DM + MC versus Control + MC, ^###^*p* < 0.001 T2DM + Pio versus T2DM + MC, ^¥¥^*p* < 0.01, ^¥¥¥^*p* < 0.001 Control + Pio versus Control + MC, one-way ANOVA, Tukey posttest (*n* = 5, mean ± SEM).

Expression levels of *ATF4*, *CHOP*, and *GRP78* genes, which are associated with ER stress, were determined in PVAT samples obtained from rat thoracic aortas. As shown in Figure 1, *ATF4* (2.95-fold), *CHOP* (2.46-fold), and *GRP78* (4.79-fold) mRNA levels were significantly increased in T2DM compared to the control group (T2DM + MC vs. Control + MC, *p* < 0.001, *p* < 0.001, and *p* < 0.01, respectively, *n* = 5). In addition, Pio treatment in the diabetes group increased *GRP78* (3.72-fold) mRNA levels further compared to the T2DM + MC group (T2DM + Pio vs. T2DM + MC, *p* < 0.001, *n* = 5); however, no change was observed in *ATF4* and *CHOP* mRNA levels. On the other hand, all these ER stress–related genes, *ATF4* (8.14-fold), *CHOP* (2.36-fold), and *GRP78* (8-fold) mRNA levels, were also determined to be increased following Pio administration in the control group (Control + Pio vs. Control + MC, *p* < 0.001, *p* < 0.01, and *p* < 0.001, respectively, *n* = 5) (Figure 1).

### 3.4. Autophagy-Related Gene Expression Levels

**Figure 2:**
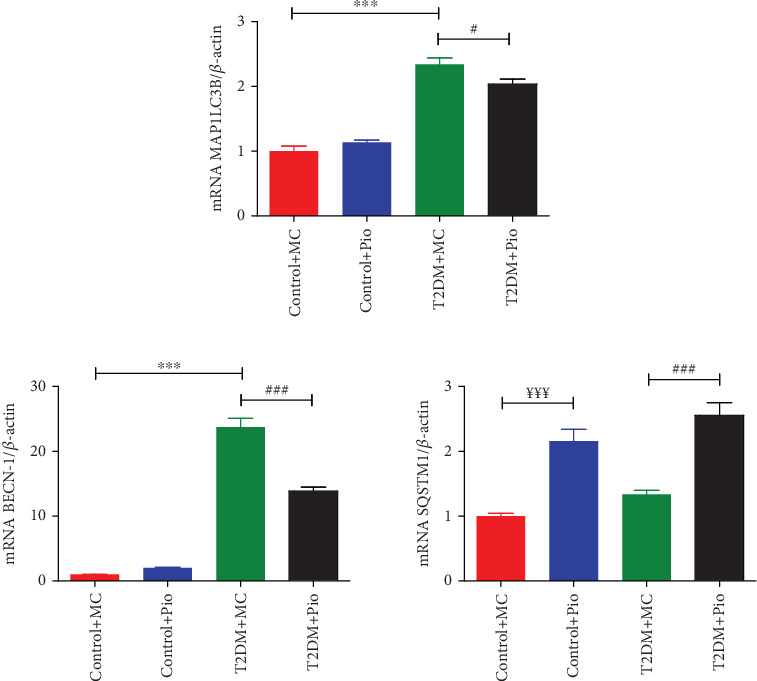
Comparison of autophagy-related *MAP1LC3B*, *BECN-1*, and *SQSTM1* gene expression levels in PVAT of control (Control + MC and Control + Pio) and diabetic (T2DM + MC and T2DM + Pio) rats. ⁣^∗∗∗^*p* < 0.001 T2DM + MC versus Control + MC, ^#^*p* < 0.05, ^###^*p* < 0.001 T2DM + Pio versus T2DM + MC, ^¥¥¥^*p* < 0.001 Control + Pio versus Control + MC one-way ANOVA, Tukey posttest (*n* = 5, mean ± SEM).

Expression levels of *MAP1LC3B*, *BECN-1*, and *SQSTM1* genes, which are associated with autophagy, were determined in PVAT samples obtained from rat thoracic aortas. As shown in Figure 2, *MAP1LC3B* (2.34-fold) and *BECN-1* (23.74-fold) mRNA levels were significantly increased in the T2DM group compared to the corresponding control (T2DM + MC vs. Control + MC, *p* < 0.001, *n* = 5), whereas no change was observed in *SQSTM1* mRNA levels. In addition, Pio administration decreased *MAP1LC3B* (13.24%) and *BECN-1* (41.58%) mRNA levels in the T2DM group compared to the corresponding control (T2DM + Pio vs. T2DM + MC, *p* < 0.05 and *p* < 0.001, respectively, *n* = 5). However, *SQSTM1* mRNA levels were increased following Pio administration in both control (2.16-fold; Control + Pio vs. Control + MC, *p* < 0.001, *n* = 5) and T2DM groups (1.91-fold; T2DM + Pio vs. T2DM + MC, *p* < 0.001, *n* = 5). Moreover, Pio administration did not modify *MAP1LC3B* and *BECN-1* mRNA levels in the control group (Control + Pio vs. Control + MC, *p* > 0.5, *n* = 5) (Figure 2).

## 4. Discussion

Pio, a widely used oral antidiabetic drug that acts through PPAR-*γ*, has many favorable effects on the cardiovascular system. PVAT plays a major role in vascular system physiology, and studies investigating the possible effect of Pio on PVAT are very limited. PVAT dysfunction occurs in pathologic conditions such as T2DM due to increased inflammation and oxidative stress. Recent studies on T2DM have demonstrated that ER stress and autophagy responses are also induced in adipose tissue, whereas the precise role of ER stress and autophagy in PVAT dysfunction remains largely unknown. Furthermore, there is no study examining the influence of Pio on ER stress and autophagy in PVAT in T2DM. In this study, we investigated the possible influence of Pio on ER stress and autophagy in the thoracic PVAT of HFD/STZ-induced T2DM rats. Pio administration increased the ER stress–related *GRP78* gene expression and reduced the autophagy-related *MAP1LC3B* and *BECN-1* gene expressions in PVAT of diabetic rats, while an increase was observed in *SQSTM1 gene* expression levels, which plays a role in autophagy as well as in adipogenesis.

Pio administration increased body weights in T2DM rats but did not cause any change in the control group. Consistent with current results, many clinical and preclinical studies with Pio showed weight gain in diabetic condition [4, 26–28]. Although increased body weight implies a worsened metabolic profile, it was demonstrated that Pio administration reduces visceral fat and changes visceral–subcutaneous fat distribution which is associated with a healthier metabolic profile [27, 29]. On the other hand, elevated nonfasting blood glucose, total cholesterol, and triglyceride levels were observed in HFD/STZ T2DM rats, correlating with clinical T2DM in terms of changes in biochemical parameters, while Pio administration reversed the increase in nonfasting glucose and triglyceride levels in T2DM, consistent with previous studies [4, 26, 30, 31].

ER stress and autophagy are also shown to be increased in T2DM in addition to oxidative stress and inflammation. Several studies have displayed increased ER stress in various organs and tissues, including adipocytes and PVAT, in T2DM [7, 32, 33]. In the present study, we showed that ER stress markers *GRP78*, *ATF4*, and *CHOP* mRNA expressions were significantly increased in aortic PVAT of HFD/STZ-induced T2DM rats, consistent with a previous study performed in aortic PVAT of diabetic (db/db) mice [7]. Originally, in the current study, Pio administration in HFD/STZ-induced T2DM rats further increased mRNA levels of *GRP78* while unchanged mRNA levels of *ATF4* and *CHOP* in aortic PVAT. To our knowledge, this is the first study investigating the effects of Pio on ER stress in PVAT. *GRP78* is known as a critical chaperone in ER stress which mitigates cellular stress and enhances protein refolding [33]. In the present study, its upregulation may reflect a role for Pio in improving cellular homeostasis despite ongoing stress in T2DM. Moreover, the lack of an effect on *ATF4* and *CHOP* mRNA levels suggests that Pio does not exacerbate proapoptotic ER stress pathways in T2DM. Considering this data, it is reasonable to suggest that Pio may influence the cellular stress in PVAT besides its primary mechanism of action in T2DM. Moreover, Pio administration also increased all the selected ER stress–related genes in the control group which suggests that it can activate ER stress pathways even in the absence of metabolic stress. Supportively, there are several studies showing that thiazolidinediones (TZDs) induce ER stress in in vitro conditions such as in human pancreatic cells [34], GN4 rat liver epithelial cells [35], and human coronary artery endothelial cells [14, 15], as well as in in vivo conditions such as rat gastric inflammation [17], nonobese diabetic [16], and obese C57BL/6J mice [18]. On the other hand, there are also studies reporting that TZDs have inhibitory effects on ER stress in both in vitro [20, 36] and in vivo [13, 19, 21] conditions, while some other studies carried out on human adipose cell line and mouse inguinal white adipocytes displayed no change in ER stress by Pio or rosiglitazone, respectively [22, 37]. Overall, according to current knowledge from the literature, the effects of TZDs, including Pio, on ER stress are controversial depending on tissue or cell types, experimental models used, and doses applied.

Increased *GRP78* mRNA expression level despite *ATF4* and *CHOP* in the aortic PVAT of HFD/STZ-induced T2DM rats treated with Pio is interesting and may also involve other mechanisms beyond ER stress. In relation, it was previously shown that *GRP78* is required for adipocyte differentiation and suggested to play an essential role in adipogenesis [38]. Concerning our findings, the remarkable increase in the expression of *GRP78* mRNA might also be associated with Pio-induced adipogenesis in PVAT of HFD/STZ-induced T2DM rats. Supportively, our recent findings demonstrated that Pio administration increased PVAT mass and adipocyte size in HFD/STZ T2DM [4].

UPR signaling stimulates autophagy in order to enhance its capacity to degrade misfolded proteins and damaged ER. In this study, expression levels of autophagy-related genes, *MAP1LC3B* and *BECN-1*, were increased in HFD/STZ-induced T2DM rats, showing elevated autophagic response in PVAT. This finding is consistent with a previous study that reports autophagy activation in adipocytes obtained from the subcutaneous adipose tissues of obese T2DM patients [5]. On the other hand, Pio treatment was shown to enhance autophagic flux in the liver of mice fed with HFD, resulting in a decrease in hepatic steatosis [23]. However, in this study, we determined that Pio administration in HFD/STZ-induced T2DM rats significantly decreased the mRNA expression levels of *MAP1LC3B* and *BECN-1* while increased *SQSTM1* expression in PVAT, suggesting a suppressed autophagy. Consistent with our findings, Lin et al. have shown that Pio treatment decreased the expressions of autophagy proteins, *Beclin-1*, *ATG12*, and *LC3BI*, in the RVLM neurons of Wistar Kyoto rats fed with a high-fructose diet [24]. Autophagy inhibition may also be a contributing factor for the persistence of inflammation as it provides the clearance of dysfunctional proteins and organelles. This suppression is likely to account for the increased TNF-*α* levels observed in the PVAT of Pio-treated HFD/STZ-induced T2DM rats [4].

Although *SQSTM1*, also known as *p62*, is an indicator of autophagic flux, previous studies demonstrated its stimulation during adipogenesis and suggested to provide a negative feedback signal to control ERK activity to prevent excessive adipogenesis and adiposity [39, 40]. Since *SQSTM1/p62* expression is induced during adipogenesis, which supports the differentiation and lipid accumulation in adipocytes, its upregulation in PVAT may be a complementary mechanism to the adipogenic effect of Pio. Moreover, an increase in *SQSTM1/p62* gene expression levels in PVAT was also detected in the control group, besides the diabetic group, following Pio administration. These findings are likely to support our recent observations, in which an increase in PVAT mass and adipocyte size was determined in both control and HFD/STZ T2DM rats [4]. Recently, *p62* has also been reported to be a master regulator of brown adipose tissue (BAT) function that regulates thermogenesis by controlling the uncoupling protein-1 (UCP1) pathway [41]. Considering that thoracic PVAT more closely resembles BAT than white adipose tissue, further studies will be intriguing to address the influence of Pio in regard to *SQSTM1/p62*-induced BAT thermogenesis in PVAT.

While the assessment of mRNA expression via RT-PCR is a well-validated and reliable method for evaluating the activation of ER stress and autophagy pathways, the study lacks corresponding protein-level analyses as a limitation. Further studies verifying the protein-level assessments of ER stress and autophagy will strengthen their critical influence on the effects of Pio in PVAT.

## 5. Conclusion

Overall, the current study provides original findings regarding the effects of Pio on ER stress and autophagy response in PVAT of HFD/STZ-induced T2DM rats. Pio induced ER stress as evidenced by increased *GRP78* gene expression while inhibited autophagic activity as reflected by decreased *MAP1LC3B* and *BECN-1* gene expression and elevated *SQSTM1/p62* gene expression. Concerning that *GRP78* and *SQSTM1* have crucial roles in adipocyte differentiation and lipid accumulation besides ER stress and autophagy, respectively, it seems reasonable to suggest that the upregulation of these gene expressions by Pio is also related with adipogenesis. Indeed, these molecular findings correlate with PVAT remodeling and provide novel insights into the effects of Pio treatment on PVAT function, structure, and biochemical properties in this experimental T2DM model. Further studies exploring the long-term effect of increased ER stress and suppressed autophagy will clarify the impact of Pio treatment on the paracrine function of PVAT function in T2DM.

## Figures and Tables

**Table 1:** Comparison of body weights and biochemical parameters between groups at the end (15th week) of the experimental period.

**Parameter**	**Control + MC**	**Control + Pio**	**T2DM + MC**	**T2DM + Pio**
Weight (g)	419.10 ± 18.09	415.64 ± 16.06	332.20 ± 14.50^∗^	416.6 ± 23.15^#^
Nonfasting glucose (mg/dL)	97.20 ± 3.02	96.60 ± 4.38	354.3 ± 20.81^∗∗∗^	154.5 ± 15.11^###^
Total cholesterol (mg/dL)	35.5 ± 5.17	31.6 ± 6.17	69.20 ± 4.03^∗∗^	68.80 ± 3.48
Triglyceride (mg/dL)	20.67 ± 4.17	23.00 ± 2.79	139.00 ± 13.74^∗∗∗^	84.00 ± 3.6^##^

⁣^∗^*p* < 0.05, ⁣^∗∗^*p* < 0.01, ⁣^∗∗∗^*p* < 0.001 T2DM + MC versus Control + MC.

^#^ *p* < 0.05, ^##^*p* < 0.01,^###^*p* < 0.001 T2DM + Pio versus T2DM + MC, one-way ANOVA, followed by Tukey posttest (*n* = 5, mean ± SEM).

## Data Availability

The data that support the findings of this study are available from the corresponding author upon reasonable request.
